# Protein Expression Redirects Vesicular Stomatitis Virus RNA Synthesis to Cytoplasmic Inclusions

**DOI:** 10.1371/journal.ppat.1000958

**Published:** 2010-06-24

**Authors:** Bianca S. Heinrich, David K. Cureton, Amal A. Rahmeh, Sean P. J. Whelan

**Affiliations:** 1 Department of Microbiology and Molecular Genetics, Harvard Medical School, Boston, Massachusetts, United States of America; 2 Program in Virology, Harvard Medical School, Boston, Massachusetts, United States of America; Stanford University School of Medicine, United States of America

## Abstract

Positive-strand and double-strand RNA viruses typically compartmentalize their replication machinery in infected cells. This is thought to shield viral RNA from detection by innate immune sensors and favor RNA synthesis. The picture for the non-segmented negative-strand (NNS) RNA viruses, however, is less clear. Working with vesicular stomatitis virus (VSV), a prototype of the NNS RNA viruses, we examined the location of the viral replication machinery and RNA synthesis in cells. By short-term labeling of viral RNA with 5′-bromouridine 5′-triphosphate (BrUTP), we demonstrate that primary mRNA synthesis occurs throughout the host cell cytoplasm. Protein synthesis results in the formation of inclusions that contain the viral RNA synthesis machinery and become the predominant sites of mRNA synthesis in the cell. Disruption of the microtubule network by treatment of cells with nocodazole leads to the accumulation of viral mRNA in discrete structures that decorate the surface of the inclusions. By pulse-chase analysis of the mRNA, we find that viral transcripts synthesized at the inclusions are transported away from the inclusions in a microtubule-dependent manner. Metabolic labeling of viral proteins revealed that inhibiting this transport step diminished the rate of translation. Collectively those data suggest that microtubule-dependent transport of viral mRNAs from inclusions facilitates their translation. Our experiments also show that during a VSV infection, protein synthesis is required to redirect viral RNA synthesis to intracytoplasmic inclusions. As viral RNA synthesis is initially unrestricted, we speculate that its subsequent confinement to inclusions might reflect a cellular response to infection.

## Author Summary

Positive-strand and double-strand RNA viruses compartmentalize their replication machinery in infected cells. This compartmentalization is thought to favor the catalysis of RNA synthesis, and sequester viral RNA molecules from detection by innate immune sensors. For the negative-strand RNA viruses that replicate in the cytoplasm, the site of RNA synthesis is less clear. Here, using a prototype non-segmented negative-strand (NNS) RNA virus, vesicular stomatitis virus (VSV), we investigated whether viral derived inclusions are sites of RNA synthesis in infected cells. Our work shows that prior to viral protein synthesis the invading viral cores synthesize mRNA throughout the host cell cytoplasm. Viral protein expression leads to the formation of intracytoplasmic inclusions that contain the viral machinery necessary for RNA synthesis and become the predominant sites of transcription. The newly synthesized viral mRNAs escape the inclusions by transport along microtubules and this facilitates their translation. Our work demonstrates that in contrast to the positive-strand and double-strand RNA viruses, VSV does not require the establishment of specialized compartments in the cytoplasm of the cell for RNA synthesis. Our findings suggest that the confinement of RNA synthesis to inclusions once infection is established may reflect a host response to infection.

## Introduction

RNA viruses that replicate within the cytoplasm often form specialized structures that are the sites of RNA replication [1]. For positive-strand RNA viruses, replication occurs on cellular membranes, including those of the endoplasmic reticulum, secretory pathway, mitochondria and other organelles [2]–[6]. Experiments with poliovirus and with flock house virus (FHV) have provided compelling evidence that the viral RNA and the non-structural proteins required for RNA replication are localized to such sites. For FHV, electron microscopy and tomographic reconstructions of spherule-like structures invaginated from mitochondrial membranes confirm that they contain the viral replication machinery [6]. Double-strand RNA viruses form phase-dense inclusions or “viral factories” to which transcription competent viral cores and the machinery required for RNA synthesis are localized [7]. In contrast to the structures formed by positive-strand RNA viruses, the double-strand RNA virus factories are not membrane bound [8]–[10]. The formation of such specialized replication compartments is thought to concentrate the viral machinery necessary for RNA synthesis and thereby favor catalysis. Compartmentalization of the replication machinery might also shield the viral RNA from detection by cytosolic innate immune sensors.

In contrast to the evidence for the role of specialized replication compartments for positive- and double-stranded RNA viruses, the exact site of RNA synthesis for non-segmented negative-strand (NNS) RNA viruses is less well characterized. Vesicular stomatitis virus (VSV), a prototype of the NNS RNA viruses, has provided many mechanistic insights into RNA synthesis for NNS RNA viruses [11]. To initiate infection, VSV delivers a transcription competent ribonucleoprotein (RNP) core into the cell [12]. This core comprises the negative-sense genomic RNA completely encapsidated by the viral nucleocapsid protein (N) and associated with the viral RNA dependent RNA polymerase [13]. The viral components of the polymerase are a 241 kDa large protein (L) and a 29 kDa accessory phosphoprotein (P) [14]. The L protein possesses all the catalytic activities required for RNA synthesis [15], including the various steps of mRNA cap addition [16]–[24] and polyadenylation [25], and the P protein serves to bridge interactions between L and the N-RNA template [26]. An L-P complex transcribes the N-RNA template into a series of mRNAs in a start-stop mode of sequential transcription [27], [28]. The polymerase also replicates the genomic RNA to yield progeny antigenomes and genomes. Replication differs to transcription in that it depends upon ongoing protein synthesis to provide the N protein necessary to encapsidate the nascent RNA [29]. *Cis*-acting signals required for RNA replication and for each step of mRNA synthesis, including cap addition and polyadenylation have been defined (reviewed in [11]), and the enzymatic activities mapped at the single amino acid level within L.

The site(s) within the cytoplasm at which VSV RNA synthesis occurs and the cellular requirements for RNA synthesis remain uncertain. For rabies virus, a related member of the *Rhabdoviridae*, pathologic specimens of infected neuronal cells identified inclusion-like structures termed Negri bodies that contain viral nucleocapsids. This led to the suggestion that such inclusions might be sites of RNA synthesis. Subsequent studies showed that Negri body-like inclusions appear to be *bona fide* sites of RNA synthesis as they contain the viral N, P and L proteins necessary for RNA synthesis as well as the mRNA products of transcription [30], [31]. That the inclusions may be active sites of synthesis rather than storage compartments was indicated by immune fluorescence (IF) microscopy using an antibody to bromodeoxyuridine which detected inclusions following transfection of cells with bromo UTP (BrUTP) [30]. This suggests that the rabies polymerase incorporated BrUTP into RNA that was actively synthesized at the inclusion-like structures. In contrast to those observations for rabies virus, for VSV it was suggested that RNA synthesis occurs throughout the cytoplasm [32]. This conclusion was also based on incorporation of BrUTP into RNA [32]. For VSV, the presence of BrUTP labeled RNA throughout the cytoplasm could, however, reflect synthesis of RNA at specific sites followed by a subsequent distribution throughout the cytoplasm. The relationship between inclusions and viral RNA synthesis remains therefore, uncertain. In addition, although experiments performed with rabies and VSV indicate that the viral polymerase can incorporate BrUTP into viral RNA, direct biochemical evidence for this is lacking.

In the present study, working with VSV, we further probed the relationship between inclusion formation and RNA synthesis. To do this, we used recombinant viruses in which P was fused to eGFP [33] or mRFP. We show that the P protein together with the N and L proteins are localized to inclusion-like structures in infected cells. By direct biochemical analysis of the products of RNA synthesis, we demonstrate that L incorporates BrUTP into viral mRNA *in vitro* as well as in cells. Imaging the location of the viral RNA synthesis machinery and the viral RNA in infected cells by fluorescent microscopy revealed that the infecting RNP can synthesize mRNA throughout the cytoplasm. Following protein synthesis, however, viral RNA synthesis appears to be restricted to inclusions. The viral mRNAs are subsequently transported away from those inclusions in a microtubule-dependent manner to facilitate translation. Our experiments show that VSV does not require a specialized site for RNA synthesis, but the viral RNA synthesis machinery is redirected to inclusions following protein synthesis.

## Materials and Methods

### Recombinant VSV expressing fluorescent P protein

Recombinant VSV expressing eGFP fused to P was previously described [33]. We generated a similar recombinant virus in which eGFP was replaced by monomeric RFP using the same strategy except oligonucleotide primers 5′-GAAAAAAACTAACAGATATCATGGCCTCCTCCGAGGACG-3′ and 5′-CTTTTGTGAGATTATCGGCGCCGGTGGAGTGGC-3′ were used to amplify the mRFP gene from pRFP-N1 (Clontech, Mountain View, CA). Recombinant virus was recovered as described previously [34].

### Generation of an anti-L antibody

Amino acids 1594–2109 of VSV L were expressed in *Spodoptera frugiperda* (Sf21) cells from a recombinant baculovirus generated by cloning the relevant portions of the L gene under the control of the polyhedrin promoter using pFASTBAC-DUAL (Invitrogen, Carlsbad, CA). An N-terminal hexa-histidine tag was introduced to facilitate L protein purification. The L protein fragment was purified by affinity chromatography on Ni-nitrilotriacetic acid-agarose (Qiagen, Valencia, CA) followed by MonoQ then MonoS ion exchange chromatography (GE Healthcare, UK). A polyclonal antiserum was obtained following immunization of a single rabbit with purified protein (Covance, Princeton, NJ). The rabbit antiserum detects full-length VSV L in infected cell lysates by Western blot (data not shown).

### Detection of RNA and proteins in cells by immune fluorescence microscopy

Imaging experiments were performed in BSR-T7, CV-1 or Vero cells. Cells were fixed with 2% paraformaldehyde for 15 min, washed twice with phosphate buffered saline (PBS) (137 mM NaCl, 2.7 mM KCl, 100 mM Na_2_HPO_4_, 2mM KH_2_PO_4_) and treated with ice-cold 100% methanol for 3 min. Cells were rinsed twice with PBS, incubated in PBSAT (1× PBS, 0.1% Triton ×100, 1% BSA), followed by PBSA (1× PBS, 1% BSA) each for 10 minutes. For RNA detection, we used a monoclonal antibody against bromodeoxyuridine conjugated to Alexa Fluor-488 (Invitrogen) at a 1∶50 dilution in PBSAT (1× PBS, 0.05% Triton ×100, 1% BSA). Cells were incubated for 1 hour at RT or 16h at 4°C, prior to detection of immune complexes using a 1∶2000 dilution of a secondary anti-mouse antibody conjugated to Alexa Fluor-488 (Invitrogen). VSV N and M proteins were detected using monoclonal antibodies 10G4 and 23H12 [35], respectively, which were kindly provided by Dr. Douglas Lyles (Wake Forest University), followed by a 1∶750 dilution of a secondary anti-mouse antibody conjugated to DyeLight 549 (Jackson ImmunoResearch Laboratories, West Grove, PA) or Alexa Fluor-488 (Invitrogen). For detection of L, we used the rabbit polyclonal antiserum at a 1∶1000 dilution followed by an anti-rabbit secondary antibody conjugated to DyeLight-649 (1∶750) (Jackson ImmunoResearch). Cellular α-tubulin was detected using a 1∶200 dilution of the monoclonal DM1A antibody (Sigma, St Louis, MO) and visualized with Alexa Fluor-594 conjugated secondary antibody (Invitrogen) at a 1∶500 dilution. Calnexin was detected using a 1∶250 dilution of a mouse anti-calnexin antibody (BD Transduction Laboratories, Franklin Lakes, NJ). GM130 was detected using a 1∶100 dilution of a mouse anti-GM130 antibody (BD Transduction Laboratories). Early endosomal antigen 1 (EEA1) was detected using a 1∶500 dilution of a mouse anti-EEA1 antibody (BD Transduction Laboratories). Secondary labeling was performed using 1∶750 dilutions of an anti-mouse antibody conjugated to DyeLight-549 (Jackson ImmunoResearch). Lysosomes and mitochondria were detected by LysoTracker and MitoTracker dyes (Invitrogen) used according to the manufacturer's instructions.

Wide-field images were acquired using a Zeiss Axioplan 2 inverted fluorescence microscope (Carl Zeiss MicroImaging, Germany) equipped with a 63× (NA 1.4) objective. Samples were excited with a Xenon lamp, and filtered emission photons were collected with a Hamamatsu Orca-HR (C4742-94) camera (Hamamatsu, Bridgewater, NJ). Confocal images were acquired using a Zeiss observer Z1 microscope (Carl Zeiss MicroImaging) fitted with a confocal spinning disk unit (Yokogawa Electric Corporation, Atlanta, GA) and a 63× (NA 1.4) objective. Excitation wavelengths were 473 nm for Alexa Fluor-488, 561 nm for Alexa Fluor-594 or DyeLight-549 and 660 nm for DyeLight-649. For 3-D acquisitions, images were captured at intervals of 0.26 µm. The X, Y, Z positions of the stage were controlled using a PZ-2000 automated stage (Applied Scientific Instrumentation, Eugene, OR). Microscope hardware was controlled with Slidebook 4.2 Software (Intelligent Imaging Innovations, Denver, CO).

### Electron microscopy

Vero cells were infected with VSV at an MOI of 3 and fixed 6 hpi with 2.5% glutaraldehyde (Electron Microscopy Sciences, Hatfield, PA) to preserve membrane integrity and 2% paraformaldehyde (Sigma) in 0.1 M sodium cacodylate buffer (pH 7.4) (Sigma) for 1h. The cells were then postfixed for 30 min in 1% osmium tetroxide (OsO_4_)/1.5% potassiumferrocyanide (KFeCN_6_) (Electron Microscopy Sciences), washed 3 times in H_2_O and incubated in 1% aqueous uranyl acetate (Sigma). This was followed by 2 washes in H_2_O and subsequent dehydration in grades of alcohol for 5 min each (50%, 70%, 95%, 2× 100%).

For immunogold EM, infected cells were fixed 6 hpi with 2% paraformaldehyde (Sigma) and labeled with primary antibodies against viral L (1∶100 dilution) and N (1∶50 dilution) as above. To detect P, we infected cells instead with VSV-eGFP-P and visualized the location of P with a rabbit anti-GFP antibody (1∶50 dilution) (Sigma). Secondary labeling was performed with anti-rabbit or anti-mouse nanogold-1.4 nm (1∶50 dilution) in 1% BSA for 1 h at RT. Samples were washed 5× in 1× PBS/1% BSA for 1h and postfixed in 1% glutaraldehyde (Electron Microscopy Sciences) in 1× PBS for 10 min. Cells were then washed 3 times for 5 min in PBS, followed by 2 washes for 5 min in deionized water and 1 wash for 5 min in 0.02 M citrate buffer. The 1.4 nm gold particles were silver enhanced (giving ∼15–40 nm particles) by incubating the samples for 4 min in freshly mixed developer using the HQ Silver Enhancement kit (Nanoprobes, Yaphank, NY) and rinsed 3 times in deionized water for 1 min. Cells were treated with 0.5% osmium tetroxide before dehydration.

For embedding, unlabeled and immunogold labeled cells were removed from dishes using propyleneoxide (Sigma), pelleted at 3000 rpm for 3 min and infiltrated for 2 h in an equal mixture of propyleneoxide and TAAB Epon (Marivac Canada Inc., St. Laurent, Canada). The samples were subsequently embedded in TAAB Epon and polymerized at 60 degrees C for 48 h. Ultrathin sections (about 60nm) were cut on a Reichert Ultracut-S microtome, picked up on to copper grids stained with lead citrate and examined in a TecnaiG^2^ Spirit BioTWIN. Images were recorded with an AMT 2k CCD camera.

### Incorporation of modified nucleotides into viral RNAs *in vitro*

Viral RNAs were transcribed *in vitro* as previously described [36] with minor modifications [37]. Detergent activated, purified recombinant VSV (rVSV) (10 µg) was incubated in the presence of nucleoside triphosphates (1 mM ATP and 0.5 mM each of CTP, GTP and UTP). Where indicated, reactions were supplemented with 0.1–1 mM 5-bromouridine 5′-triphosphate sodium salt (BrUTP) (Sigma), fluorescein-12-UTP, -GTP, -ATP, Alexa Fluor-488-UTP (Invitrogen), Cy3-17-UTP (General Electric Life Sciences, UK) or 15 µCi of [α-^32^P]-GTP (Perkin Elmer, Waltham, MA). As a control, transcripts were also synthesized by T7 RNA polymerase (New England Biolabs, Beverly MA) using the previously described VSV expression plasmid pN [38].

### Incorporation of BrUTP into RNA in cells

Approximately 30,000 BSR-T7 cells grown on cover slips in 24 well plates were infected with VSV at the specified MOI (3–500). At the indicated times post infection, cells were depleted of uridine by low glucose DMEM (Invitrogen) supplemented with 20 mM glucosamine (Sigma), and transfected 1h later with 5 mM BrUTP in 250 µl of DMEM supplemented with 6 µl of lipofectamine 2000 (Invitrogen). In some experiments, cells were treated 15–40 minutes prior to BrUTP labeling with a variety of chemical inhibitors (Sigma). Specifically, we used 10 µg ml^−1^ actinomycin D (ActD) to inhibit cellular transcription, 100 mM nocodazole (Noc) to disrupt microtubules or 10 µg ml^−1^ puromycin (Pur) to inhibit protein synthesis. For pulse-chase analyses, the cell culture medium was supplemented with 50 mM uridine (Sigma) throughout the chase period. In some experiments, RNAs were simultaneously metabolically labeled by the incorporation of 33 µCi ml^−1^ [^3^H]-uridine (Perkin Elmer) from 4–9 hpi.

### Purification and analysis of RNA by electrophoresis

The products of *in vitro* synthesis reactions were purified using an RNeasy kit (Qiagen). For cellular RNA analysis, cytoplasmic extracts were prepared and RNA was purified by phenol-chloroform extraction as described previously [38]. Where indicated, RNAs were immune precipitated by incubation with a monoclonal antibody raised against bromodeoxyuridine (Roche Diagnostics, Indianapolis, IN). Immune precipitations were performed in Rose lysis buffer (1% Nonidet P40, 66 mM EDTA, 10 mM Tris-HCl pH 7.4) and the immune complexes collected using protein G magnetic beads (NEB). RNA was analyzed by electrophoresis on agarose-urea gels [39] and detected using a Typhoon 9400 PhosphoImager (GE Healthcare).

### Metabolic labeling of proteins

At the indicated times post infection, cells were starved of L-methionine and L-cysteine for 1h in the presence of 10 µg ml^−1^ ActD. Where indicated, cells were exposed to 10 µg ml^−1^ Pur for 1 h or 100 mM Noc during the last 15 min of starvation. Proteins were labeled by addition of 17.5 µCi [^35^S]EasyTag express (Perkin Elmer) in DMEM lacking L-methionine and L-cysteine (Invitrogen). Where indicated, nocodazole was washed out to permit repolymerization of microtubules. Total cytoplasmic proteins were analyzed by 10% SDS-PAGE and detected by phosphoimage analysis. Quantitative analyses were performed using ImageQuant Software (GE healthcare).

## Results

### In infected cells, the VSV replication machinery is found in inclusions

**Figure 1:**
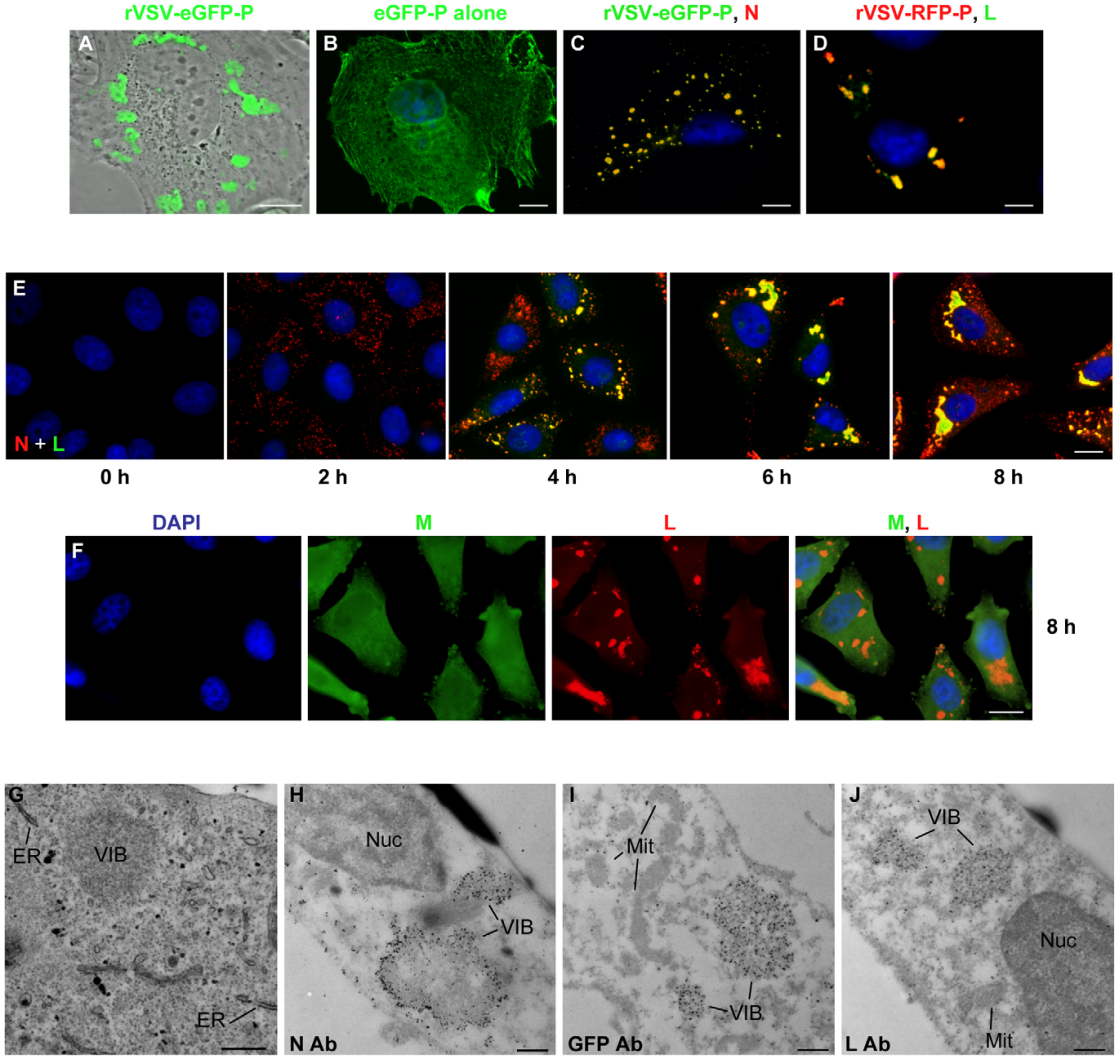
VSV N, P and L proteins localize to inclusions in infected cells. (**A**) CV-1 cells were infected with VSV-eGFP-P at an MOI of 3 and fluorescent microscopy images acquired at 5 hours post infection (hpi). (**B**) CV-1 cells were transfected with 3.8 µg of a plasmid expressing eGFP-P and examined by fluorescence microscopy at 24 hours post transfection. An image of a single representative cell illustrating the typical distribution of P is shown. (**C and D**) BSR-T7 cells were infected with VSV-eGFP-P or VSV-RFP-P respectively as in panel A, and N (panel C, red) and L (panel D, green) were detected by immune fluorescence microscopy. (**E and F**) Vero cells were infected with rVSV at an MOI of 5, and the distribution of the N (red) and L (green) proteins (panel E) or M (green) and L (red) proteins (panel F) was detected by IF microscopy at the indicated hpi. (**G**) Vero cells were infected with rVSV as in panel A and at 6 hpi were prepared for thin-section electron microscopy. To preserve intracellular membrane structures, samples were fixed with glutaraldehyde as in methods. (**H–J**) Vero cells were infected with rVSV (H, J) or rVSV-eGFP-P (I) and prepared at 6 hpi for immuno gold labeling as described in methods. Size bars are 5µm (panels A–D), 10µm (panels E, F) and 0.5µm (panels G–J). Note samples B–F were also stained with DAPI to visualize the nuclei. ER = endoplasmic reticulum, Nuc = nucleus, Mit = mitochondria, VIB = viral inclusion body.

Previously we described a recombinant VSV in which eGFP was fused to the N terminus of P [33]. In cells infected with this virus, we observed that the eGFP-P protein localized to discrete inclusions that were heterogeneous in size and shape (Figure 1A). This was not simply a consequence of protein overexpression, as we observed that eGFP-P was distributed throughout the cell when expressed alone from a plasmid (Figure 1B). The eGFP-P inclusions are visually similar to inclusions observed in rabies virus infected cells that were shown to be sites of RNA synthesis [30]. Consistent with the experiments with rabies virus, the VSV N and L proteins also colocalize with P at inclusions (Figure 1C and D respectively). The kinetics of VSV replication are very rapid in cell culture with yields of virus increasing by >2 log by 4 hour post inoculation. We therefore monitored the kinetics of inclusion formation in cells over time. To do this, we infected cells with rVSV at a multiplicity of infection (MOI) of 5 and monitored the location of the N and L proteins by IF microscopy. Multiple foci of N were detected as early as 2 hours post infection (hpi), with characteristic inclusion-like structures being visualized by 4 h (Figure 1E). As infection progressed the size of the inclusions appeared to increase (Figure 1E). In contrast to the viral proteins required for replication, the matrix (M) protein was neither enriched nor excluded from these structures (Figure 1F).

To examine the cellular location of the inclusion-like structures, we performed electron microscopy of cells infected with VSV. As previously [40], viral inclusion bodies (VIB) were detected in the cytoplasm of the cell (Figure 1G–J). These inclusions do not appear to be associated with a cellular membrane or specific organelle (Figure 1G). Consistent with this, we did not detect colocalization of the inclusions with markers for the endoplasmic reticulum, Golgi, endosomes, lysosomes, and mitochondria (Figure S1). Rather, the inclusions contain the viral N, P and L proteins which were readily detected by immunogold electron microscopy (Figure 1H–J). These observations confirm that like rabies virus, the VSV replication machinery is found in discrete viral derived inclusion-like structures in infected cells.

### Incorporation of BrUTP into viral RNA

**Figure 2:**
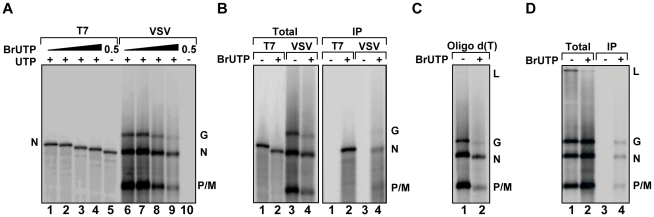
VSV RNA polymerase incorporates BrUTP during transcription *in vitro* and *in vivo*. (**A**) Incorporation of BrUTP into RNA synthesized *in vitro*. An autoradiograph of an acid agarose-urea gel is shown, depicting RNA transcribed by T7 RNA polymerase from a plasmid encoding VSV N (lanes 1–5) or synthesized by detergent activated virus *in vitro* (lanes 6–10) in the presence of increasing concentrations (0, 0.1, 0.5 or 1 mM) of BrUTP. The products of the reactions are indicated alongside the gel. (**B**) The samples of panel A were immune precipitated using an antibody raised against bromodeoxyuridine prior to acid-agarose gel electrophoresis. (**C**) The samples of panel A were isolated by oligo-dT chromatography prior to acid-agarose gel electrophoresis. (**D**) BSR-T7 cells were infected with wild-type VSV and, where indicated (+), transfected 4 hpi with BrUTP (5mM final concentration). Cells were exposed to [^3^H]-uridine for 5 hours and RNA was isolated prior to acid-agarose gel electrophoresis. Where indicated (IP) the RNA was immunoprecipitated as in panel (B).

To visualize *de novo* synthesis of viral RNA, we tested the ability of purified VSV L protein to incorporate fluorescent nucleotides *in vitro*. Viral RNA synthesis was inhibited in reactions containing fluorescein-12-UTP, -GTP or -ATP, Alexa Fluor-488-UTP or Cy3-17-UTP or the RNA products were not fluorescent (data not shown). This result indicates that L cannot incorporate nucleotides that contain such large modifications. To test whether nucleotides with smaller modifications can be incorporated into viral RNA, we supplemented *in vitro* transcription reactions performed in the presence of [^32^P]-GTP with 5-BrUTP, and monitored the products of RNA synthesis by electrophoresis on acid-agarose gels (Figure 2A). As the concentration of BrUTP in the reaction increased from 0–1 mM, the overall yield of RNA decreased and the transcripts migrated with a slightly faster mobility. The altered mobility of the RNA suggests that L incorporates BrUTP into the mRNA as a similar mobility shift is observed for transcripts synthesized by T7 RNA polymerase (Figure 2A). The presence of BrUTP in the viral transcripts was confirmed by their selective immune precipitation with an antibody directed against bromodeoxyuridine, which failed to precipitate unmodified RNA (Figure 2B). The BrUTP labeled mRNAs were also retained by oligo dT chromatography, which demonstrates that the mRNAs are full-length and contain polyadenylate (Figure 2C). The agarose-urea gels separate products based upon their molecular weight as well as charge [39], which likely accounts for the observed mobility shift.

To examine whether BrUTP is similarly incorporated into viral RNA in cells, we transfected 5 mM BrUTP into BSR-T7 cells that were infected 6 hours earlier with VSV. Infected cells were subsequently exposed to [^3^H]-uridine in the presence of ActD to permit the labeling of viral RNA, and the total cellular RNA was extracted, purified and BrUTP incorporation determined by immune precipitation prior to electrophoresis on acid-agarose gels. Consistent with the incorporation of BrUTP by the VSV polymerase *in vitro*, viral mRNAs were immune precipitated from cells that were transfected with BrUTP, but not from cells that lacked BrUTP (Figure 2D). This set of experiments demonstrates that VSV L incorporates 5-BrUTP into viral mRNA *in vitro* and in infected cells.

### Visualization of viral RNA in infected cells

**Figure 3:**
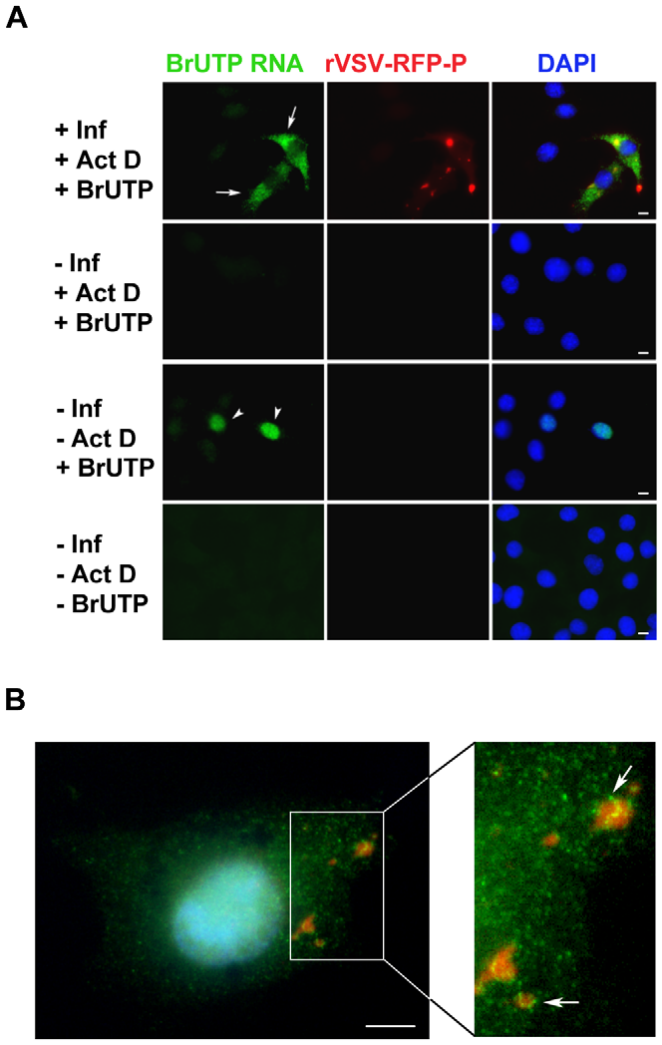
Visualization of viral RNAs in VSV infected cells. (**A**) Fluorescent microscopy images of BSR-T7 cells showing virus infection (red), viral RNA (green) and the cell nuclei (blue). Cells were infected (+Inf) with rVSV-RFP-P at an MOI of 3 or mock infected (−Inf). At 5 hpi, cells were depleted of UTP, treated with actinomycin D (+ActD) and where indicated transfected 1h later with 5mM BrUTP (+BrUTP). Following 1h incubation at 37°C to allow incorporation of BrUTP into RNA, cells were fixed and the RNA was detected using an Alexa Fluor-488 conjugated antibody against bromodeoxyuridine. The RFP-P protein was visualized at 561nm, and the cell nuclei were stained with DAPI. (**B**) Cells were infected and processed as in panel A, except that the duration of the BrUTP labeling was reduced to 30 minutes. Size bars = 5µm.

To visualize the cellular localization of viral RNA, we infected BSR-T7 cells with rVSV-RFP-P, and 5 hours later treated the cells with ActD to inhibit cellular transcription and glucosamine to deplete the intracellular pool of uridine [41], [42]. Following a 1 hour incubation, the RNA was labeled by incorporation of BrUTP for 1 hour and was subsequently visualized by IF microscopy. In infected cells - as evidenced by the RFP-P inclusions - we found BrUTP labeled RNA distributed throughout the cytoplasm (Figure 3A, row 1, arrows). No BrUTP labeled RNA was detected in uninfected cells (Figure 3A, rows 1 and 2). As expected, in the absence of ActD we observed BrUTP labeled cellular RNA, which was predominantly localized to the nucleus (Figure 3A, row 3, arrowheads), and no RNA was visualized in cells that did not receive BrUTP (Figure 3A, row 4). This result shows that VSV RNA is localized throughout the cytoplasm in infected cells. We could not discriminate, however, whether viral RNA was synthesized throughout the cytoplasm, or at the RFP-P inclusions followed by subsequent movement. Consistent with this latter idea, we detected BrUTP labeled RNA in close proximity to inclusions as well as throughout the cytoplasm when BrUTP incorporation was allowed to proceed for only 30 minutes prior to fixation (Figure 3B, arrows).

### Viral RNAs are transported away from their site of synthesis in a microtubule- dependent manner

**Figure 4:**
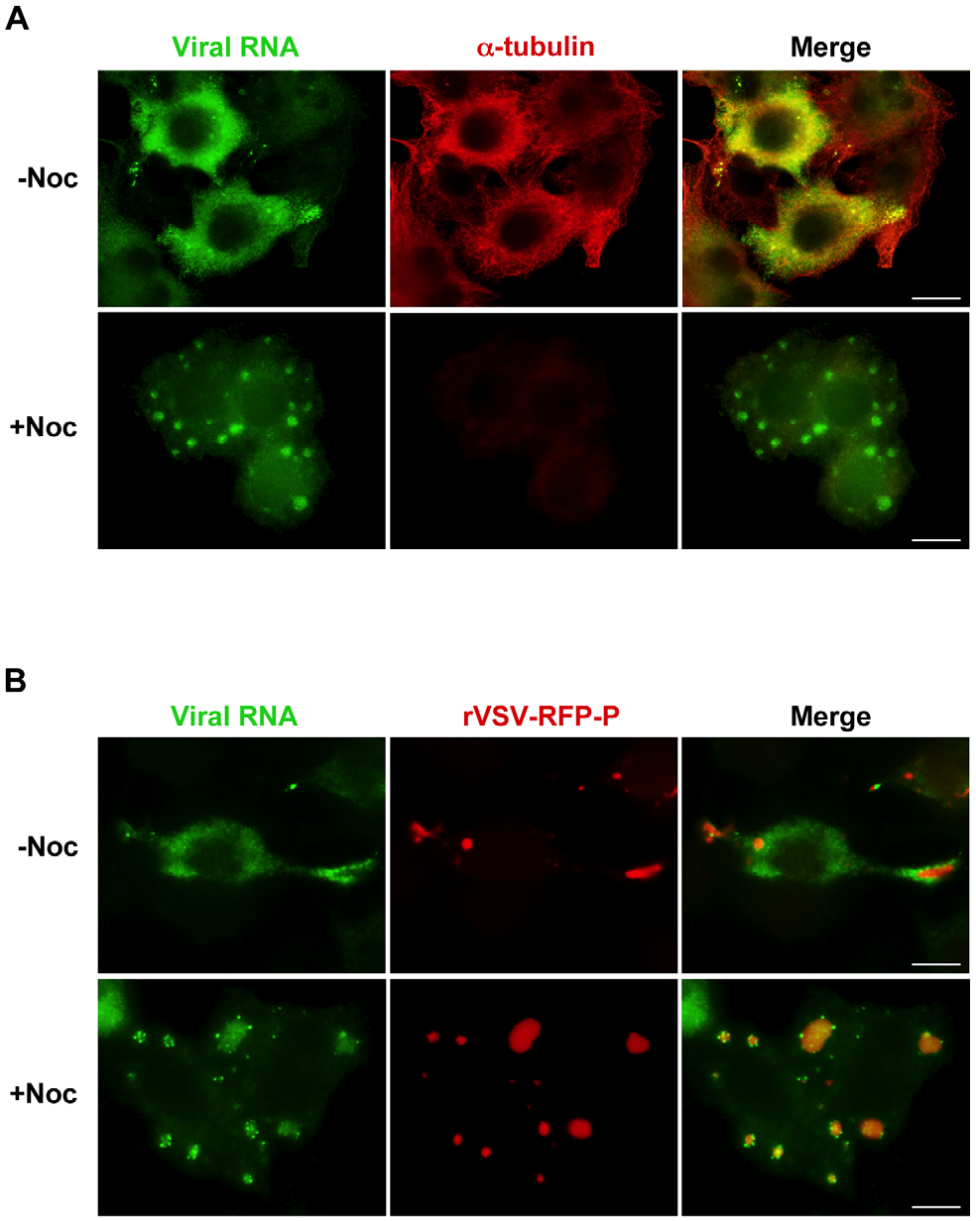
Viral RNA concentrates at the inclusions following depolymerization of microtubules with nocodazole. (**A**) rVSV infected BSR-T7 cells (MOI = 3) were treated 4 hpi with nocodazole for 1h before transfection with BrUTP. Disruption of the microtubule network was confirmed by immunostaining with an α-tubulin antibody (red). Viral RNAs (green) were visualized as described in Figure 3. (**B**) BSR-T7 cells were infected with rVSV-RFP-P (red), treated with nocodazole and examined by fluorescence microscopy as in (A). Size bars = 10µm.

Movement of viral RNA from inclusions may occur *via* a passive or an active transport mechanism. The process of active transport should be dependent upon the presence of an intact cytoskeletal network. To examine whether the distribution of viral RNA is microtubule-dependent, we monitored RNA localization in VSV infected cells following chemical depolymerization of microtubules (MTs) with nocodazole. Under those conditions viral RNA was confined to specific regions of the cytoplasm (Figure 4A). In VSV-RFP-P infected cells, we observed the viral RNA surrounding the RFP-P inclusions in discrete quanta following a 40-minute pulse of BrUTP (Figure 4B, lower panel). These images suggest that viral RNA synthesis occurs at the inclusions, and that viral RNA is transported away from the inclusions in a MT-dependent manner.

### A pulse-chase analysis confirms that viral RNA is transported away from inclusions

**Figure 5:**
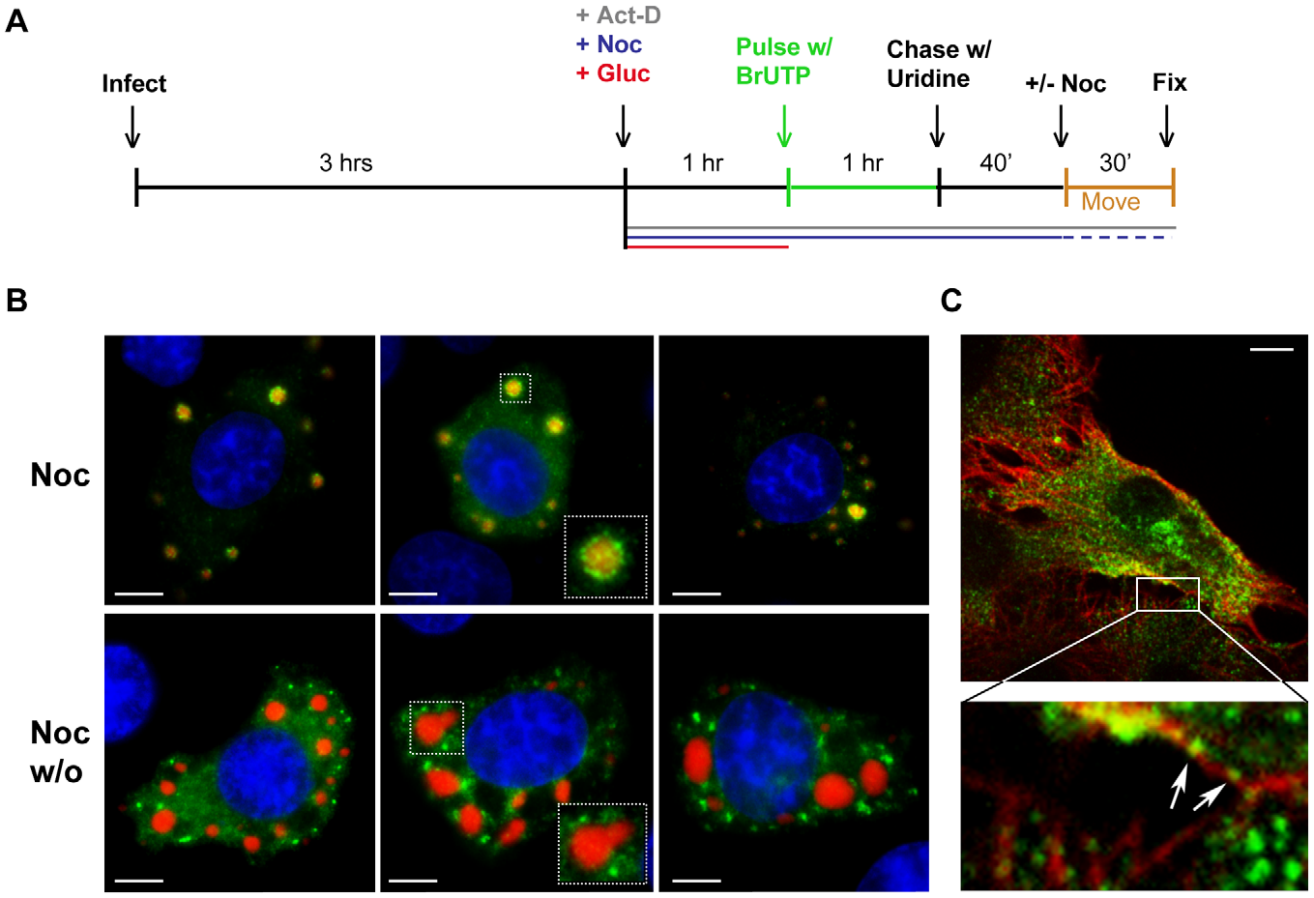
Viral RNA is transported away from inclusions in a microtubule-dependent manner. (**A**) Schematic of the RNA pulse-chase experiment performed in (B). (**B**) BSR-T7 cells were infected with rVSV-RFP-P (MOI = 3) and RNA was labeled using the strategy outlined in A. Viral RNAs (green) and RFP-P (red) are shown following a 70-minute chase in which nocodazole was present (upper panels) or washed out (w/o, lower panels) for the last 30 min. (**C**) An image of an rVSV infected BSR-T7 cell showing some BrUTP labeled RNA (green) is associated with α-tubulin (red). Size bars = 5µm.

To confirm that viral RNA was transported away from inclusions, we performed a pulse-chase analysis. To do this, we first depleted intracellular pools of uridine with glucosamine (+Gluc) [41], [42], labeled the RNA by incorporation of BrUTP and then subsequently “chased” with a 10-fold excess of unlabeled uridine (see schematic in Figure 5A). When nocodazole was absent during the indicated chase period, the viral RNA granules were found a range of distances away from the inclusions rather than closely surrounding them (Figure 5B). This observation confirmed that the RNA was transported away from the inclusions in a microtubule-dependent manner and suggests that this is an active process. Consistent with this notion, RNA granules were observed along and in close proximity to microtubules (Figure 5C, enlarged inset, arrows). These RNA localization experiments reveal that VSV RNA is synthesized at inclusions in infected cells and that the viral RNA is transported away from those inclusions in a microtubule-dependent manner to become distributed throughout the cytoplasm.

### The viral replication machinery is localized to inclusions that are active sites of RNA synthesis

**Figure 6:**
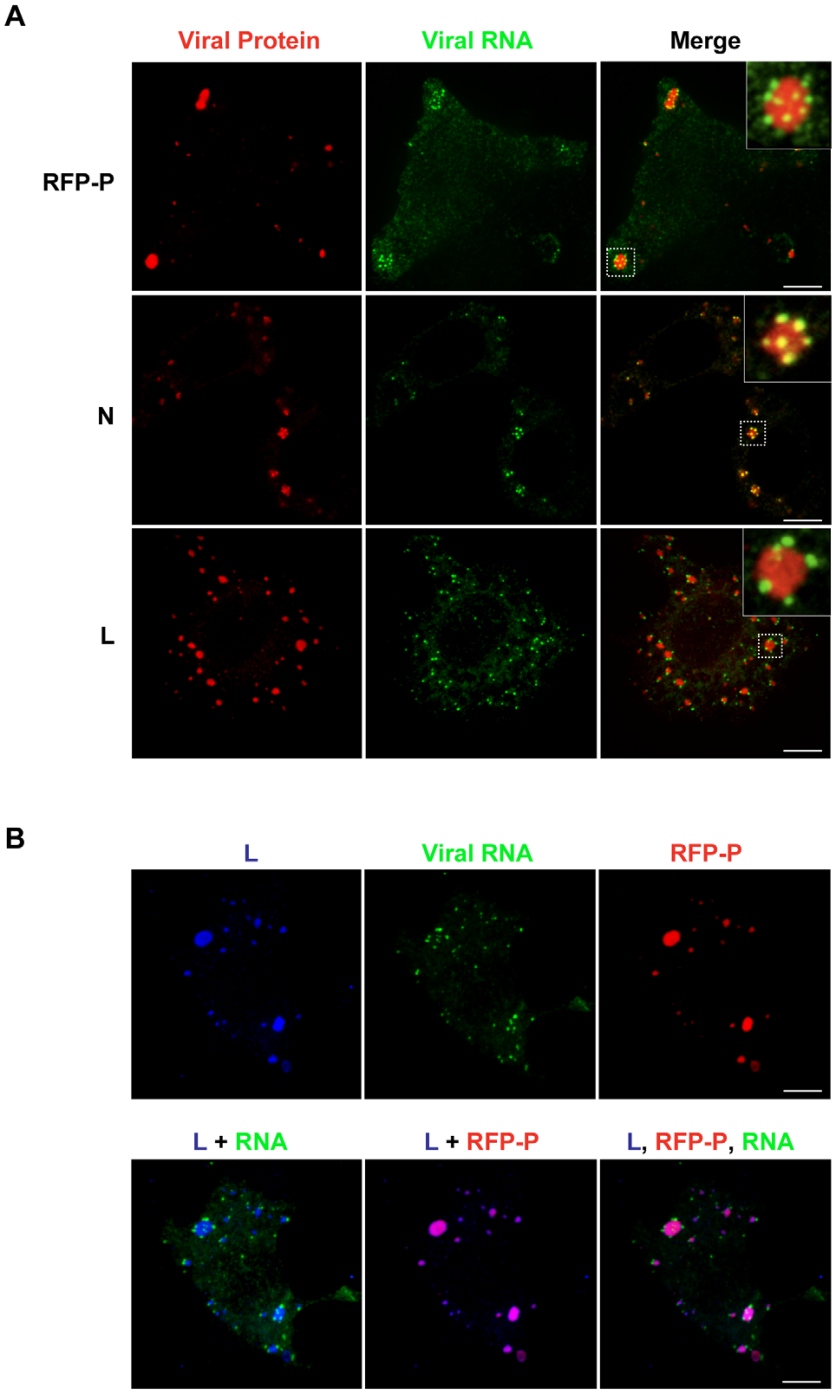
The VSV N, P and L proteins localize at the sites of RNA synthesis. (**A**) Images of BSR-T7 cells infected with rVSV or rVSV-RFP-P showing the distribution of the viral replication machinery N, P and L (red) in relation to newly synthesized VSV RNA (green). Cells were infected at an MOI of 3 and at 4 hpi depleted of intracellular UTP and treated with ActD and nocodazole. Following a 1h incubation, cells were transfected with BrUTP and fixed 40 minutes later. N and L proteins and viral RNA were detected by antibody staining and images acquired by confocal microscopy. (**B**) Cells were treated as above. Triple labeling shows viral RNA (green) at the site of synthesis together with P (red) and L protein (blue). Size bars = 5µm.

The viral protein requirements for RNA synthesis are N, P and L. To determine whether inclusions containing N, P and L are active sites of RNA synthesis, we infected cells with either rVSV or rVSV-RFP-P and visualized RNA and protein using confocal microscopy. In these experiments, we restricted RNA to its site of synthesis by treating cells with nocodazole prior to transfection of BrUTP. The viral RNA was observed as granular structures around inclusions that were visualized by RFP-P expression or following staining with antibodies against N or L (Figure 6A). All visible inclusions are decorated with viral RNA suggesting that they are each sites of RNA synthesis (Figure 6A and Videos S1, S2, S3, S4, S5 and S6). Triple wavelength imaging of the RFP-P, L and the BrUTP RNA confirmed that the viral proteins colocalize and that viral RNA is present at the inclusions (Figure 6B). This experiment demonstrates that the viral protein requirements for RNA synthesis are localized to inclusions in infected cells, and that those inclusions are sites of RNA synthesis. Although the N, P and L proteins colocalize to inclusions, the RNA surrounds, but appears to be excluded from, the inclusions. Whether this reflects synthesis of the RNA at specific sites on the surface of the inclusion or a limitation of detection of the RNA within the inclusion is uncertain. The RNA decorating the inclusion also colocalized with N protein, but not the P or L protein. This colocalization with N, may reflect the previously reported association of viral mRNA with N protein [43], and/or may represent the N encapsidated viral genomes.

### Primary RNA synthesis occurs throughout the cytoplasm

**Figure 7:**
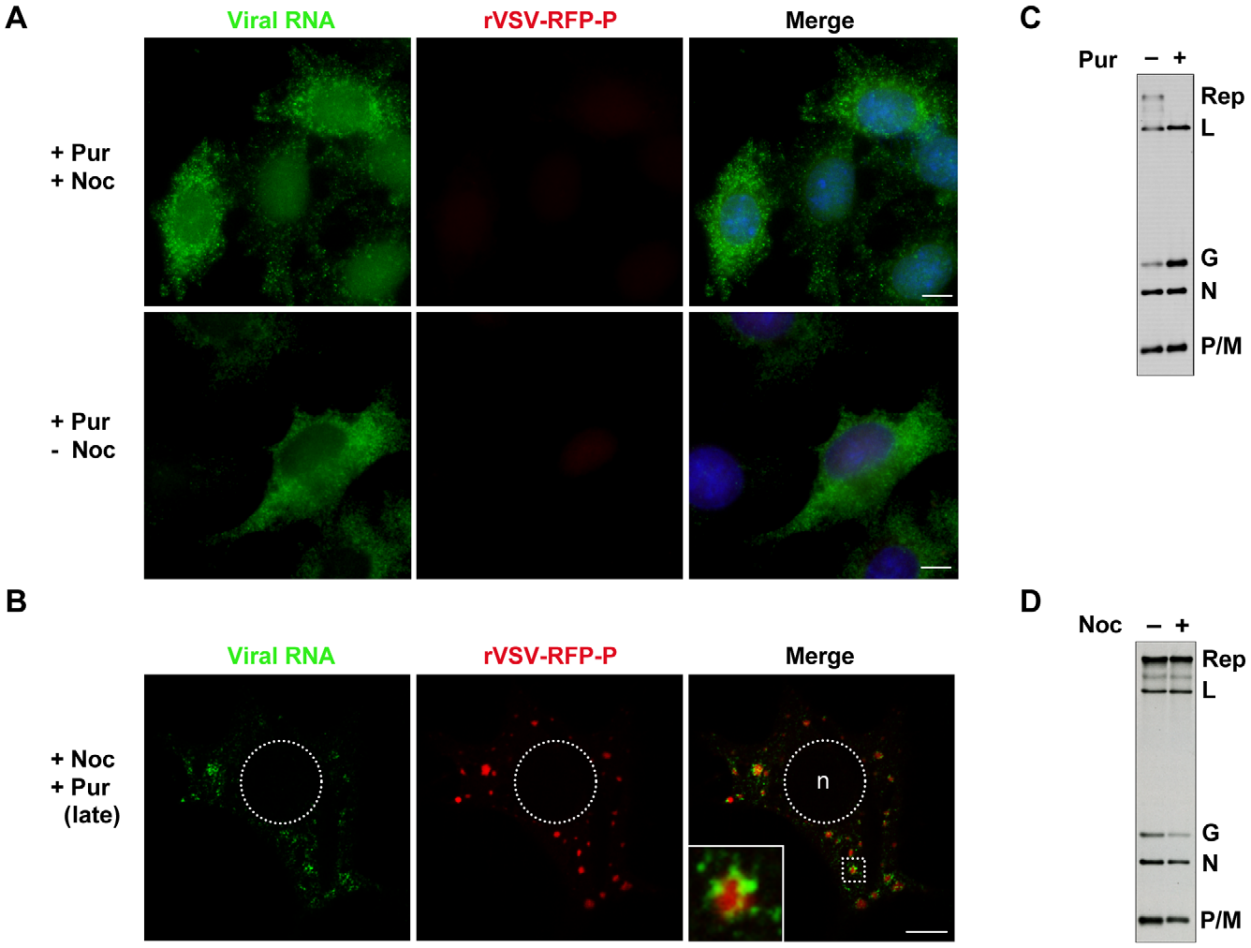
Viral protein synthesis is required to establish inclusions that are sites of RNA synthesis. (**A**) Visualization of primary mRNA transcripts. BSR-T7 cells were treated with puromycin 15 minutes before infection with rVSV-RFP-P (red) at an MOI of 500. At 2 hpi, cells were depleted of intracellular UTP, exposed to ActD and where indicated, treated with nocodazole. After 1 h, cells were transfected with BrUTP and viral RNA (green) was detected 1h later by immune fluorescence microscopy. (**B**) Visualization of secondary mRNA transcription. Cells were infected with rVSV-RFP-P at an MOI of 3, treated with puromycin at 7hpi, depleted of UTP, followed by treatment with nocodazole and ActD. Cells were then transfected with BrUTP and RNA detected 40 minutes later by immune fluorescence microscopy. Note, the size bars in panels A and B = 5µm. (**C and D**) BSR-T7 cells were infected with rVSV at an MOI of 3 and viral RNA labeled with [^3^H]-uridine as described in methods, resolved by electrophoresis on acid-agarose gels and visualized by fluorography. Where indicated, cells were pretreated for 15-minutes with puromycin (panel C) or nocodazole (panel D) which remained present throughout the course of the labeling.

The above experiments show that viral RNA is synthesized at, and actively transported away from inclusions. To establish infection however, the input RNP must synthesize mRNA presumably in the absence of such inclusions. To determine where such primary transcription occurs, we infected BSR-T7 cells with rVSV-RFP-P at an MOI of 500 in the presence of the protein synthesis inhibitor puromycin and monitored RNA synthesis by BrUTP incorporation. Genome replication requires the ongoing synthesis of N protein [29], so treatment of cells with puromycin results exclusively in mRNA synthesis. Under those conditions, viral mRNA was distributed throughout the cytoplasm even when active transport on microtubules was abolished by treatment with nocodazole (Figure 7A). This observation suggests that protein synthesis is required for inclusion formation at which subsequent RNA synthesis occurs, and demonstrates that the viral mRNAs are not simply restricted to specific cytoplasmic sites by disruption of the MT network. By infecting cells with rVSV and detecting the input RNPs and primary transcripts we also show that they are distributed throughout the cytoplasm at distinct locations (Figure S2). This distribution of mRNA throughout the cytoplasm is not simply a consequence of inhibiting protein synthesis, as treatment of cells with puromycin at 7 hpi results in the typical distribution of mRNA around inclusions (Figure 7B). By metabolic labeling of viral RNA, we confirmed that puromycin inhibits genome replication (Figure 7C). In contrast, nocodazole treatment is relatively inert with regard viral RNA synthesis (Figure 7D). Taken together these data show that primary viral transcription occurs throughout the cytoplasm, and that protein synthesis is required to establish an inclusion at which subsequent RNA synthesis takes place. Since genome replication is inhibited in the presence of the protein synthesis inhibitor puromycin, the experiment also directly demonstrates that the inclusions are sites of secondary mRNA synthesis.

### Microtubule-dependent transport of the viral mRNA enhances translation

**Figure 8:**
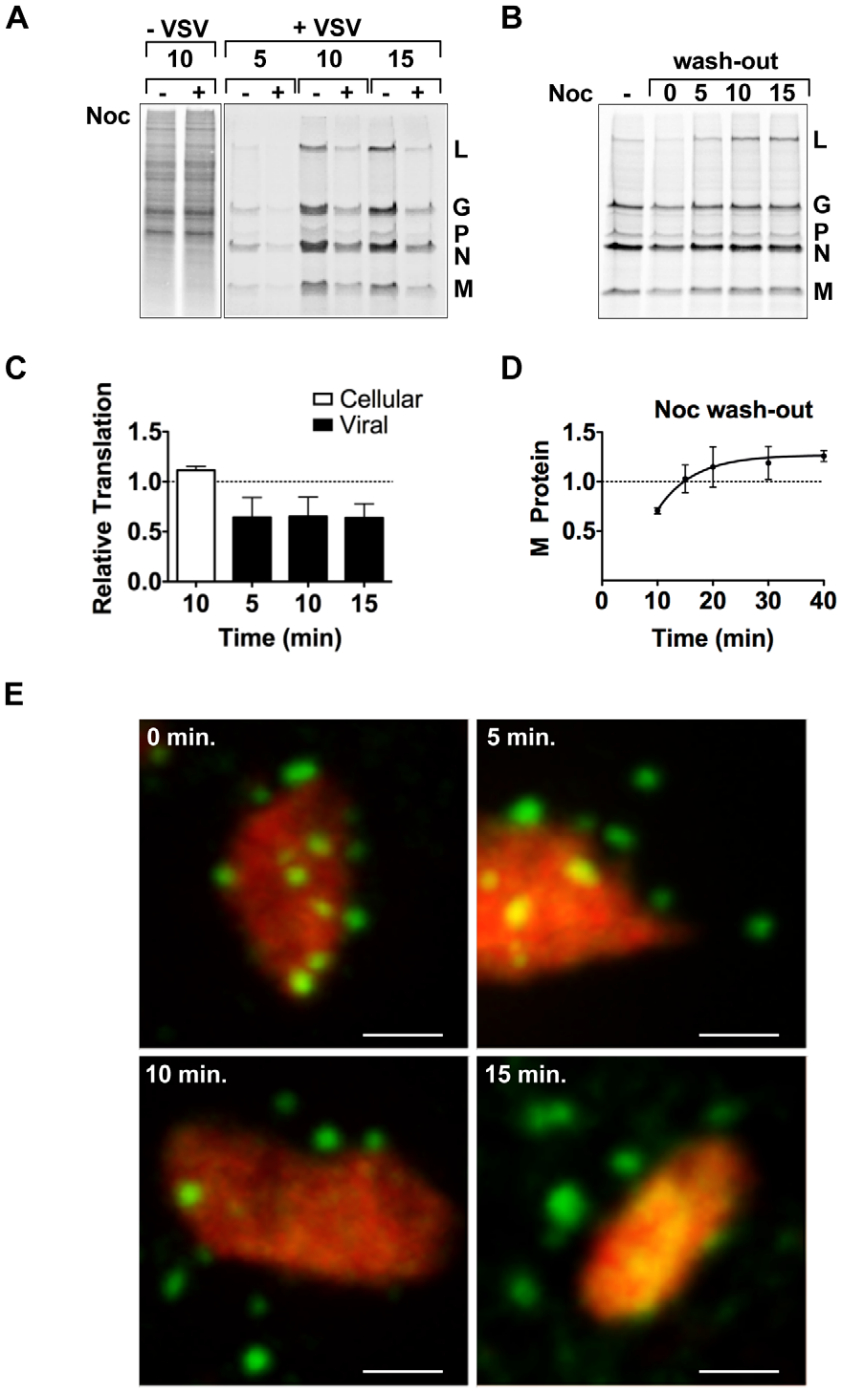
Microtubules are required for efficient translation of VSV mRNAs. (**A**) BSR-T7 cells were infected with rVSV at an MOI of 3 and following a 15 minute treatment with nocodazole 4 hpi, proteins were labeled by metabolic incorporation of [^35^S]-methionine. After a 5–15 minute labeling period, cytoplasmic extracts were prepared and analyzed by SDS-PAGE and proteins detected using a phosphoimager. (**B**) BSR-T7 cells were treated as in (A) except that nocodazole was washed out (wash-out) from the media prior to metabolic labeling. [^35^S]-methionine was added immediately after removal of nocodazole (time point 0) or at subsequent 5-min intervals, and incorporation allowed to proceed for 10 min. (**C**) Quantitative analysis of protein synthesis from four independent experiments of panel A. The intensity of the entire lane (for cellular translation) or of the M protein (for viral translation) was measured using ImageQuant software and expressed as a % of the intensity of the time matched control that lacked nocodazole. Error bars represent standard deviations. (**D**) Quantitative analysis of protein synthesis from three independent experiments of panel B. The intensity of the M band for each time point was compared to the –Noc control. Error bars represent standard deviations. (**E**) BSR-T7 cells were infected with rVSV-RFP-P at an MOI of 3. At 4 hpi, cells were exposed to ActD and nocodazole, and 15 minutes later were transfected with BrUTP. Following 40 min of BrUTP incorporation, nocodazole was removed from the media and cells were fixed immediately (0 min) or following a 5–15 min incubation. RNA and inclusions were visualized as previously. Size bar = 3 µm.

To determine whether the transport of the viral mRNA was biologically important, we evaluated the effect of nocodazole treatment on the rate of viral protein synthesis by metabolic labeling. Following short-term nocodazole treatment, the rate of viral protein synthesis was diminished by 40% compared to that in untreated cells (Figure 8A and C). In contrast, the rate of total cellular translation was unaffected by nocodazole treatment (Figure 8A and C). This suggests that a MT-dependent transport process is specifically required for efficient viral protein synthesis. To correlate those effects on protein synthesis with transport of the VSV mRNA, we performed an experiment in which we washed out nocodazole and metabolically labeled cells with [^35^S]-methionine (Figure 8B and D) or monitored the location of the BrUTP RNA (Figure 8E). Translation of viral mRNA was rapidly restored (within 15 minutes) of nocodazole wash-out (Figure 8B and D), and this restoration of protein synthesis capability was congruent with the transport of the mRNA away from the inclusion (Figure 8E). While full assembly of the microtubule network takes longer than the time period of our labeling experiment, the repolymerization of microtubules is visible within 5–10 minutes of nocodazole wash-out in many fibroblast cells, including Vero and baby hamster kidney cells used here [44], [45]. This experiment therefore correlates the effects of nocodazole treatment on protein synthesis with the physical location of the mRNA and provides evidence that the transport of the viral mRNA away from inclusions is required to maintain a high rate of protein synthesis.

## Discussion

In this study, we examined the sites of viral RNA synthesis in cells infected with VSV. By direct biochemical analysis of the products of RNA synthesis *in vitro* and in cells we show that VSV L incorporates BrUTP into viral mRNA. We used triple wavelength confocal microscopy to image the localization of the viral mRNA and the N, P and L proteins that are necessary for RNA synthesis. The following major conclusions are apparent from our study. The incoming viral RNP can synthesize mRNA throughout the cytoplasm of the cell. Once protein synthesis occurs, viral inclusions form that contain the viral RNA synthesis machinery and are the major sites of RNA synthesis. For efficient translation the viral mRNAs are transported away from inclusions. These findings are directly relevant to understanding whether viral derived inclusions represent preferred sites of RNA synthesis established by viruses in cells, or are instead a secondary consequence of the host response to infection.

### Location of VSV RNA synthesis

Detection of the first RNA synthetic events even following high multiplicity infection is challenging to visualize. All studies to date have reported on the presence of sites of RNA synthesis once replication has been established. Here we took advantage of the intrinsic properties of VSV with regard the ability to infect cells at high MOI, and inhibit all RNA synthesis other than that directed by the input genomic RNP complex. By performing infections in the presence of protein synthesis inhibitor puromycin, we show that primary viral mRNA synthesis occurs throughout the cytoplasm (Figure 7). The distribution of the primary mRNAs appears unaffected by nocodazole treatment of cells (Figure 7A), consistent with the idea that the infecting RNP can synthesize RNA anywhere within the cytoplasm and that a specialized site is not required to compartmentalize the RNA synthesis machinery. In contrast, once viral protein synthesis occurs, RNA synthesis appears to be predominantly localized to inclusions. Although our experiments demonstrate that protein synthesis is essential for the formation of the inclusion, we cannot be certain whether this reflects a requirement for viral protein synthesis alone, or whether cellular protein synthesis might also be required. The requirement for viral protein synthesis raises the possibility that the formation of such inclusions may reflect an ability of the host cell to detect the “foreign” viral proteins, which triggers a response that results in the viral replication machinery being corralled into an inclusion-like structure. Such an idea is also compatible with the observation that inclusions are not observed until viral replication is established (Figure 1E).

Conversely, viral proteins might be specifically targeted to such inclusions to promote RNA synthesis and/or assembly of progeny RNPs. While the input RNP can synthesize mRNA in the absence of inclusions (Figure 7 and S2), the picture for genome synthesis is not certain. The kinetics with which inclusions are detected in cells (Figure 1E) suggests that genome replication might occur in the absence of inclusion formation. We cannot, however, eliminate the possibility that smaller inclusions that are not readily visualized by our microscopy approaches are present prior to genome replication. Once inclusions are formed, they become the major sites of RNA synthesis. Although we did not formally demonstrate that RNA replication itself occurs at the inclusion, viral genomes must be present at the inclusion to provide the template for mRNA synthesis. The simplest interpretation of the data is that replication as well as transcription occurs at the inclusions.

Experiments with the related NNS RNA virus, parainfluenza viurs 5 (PIV5) show that viral genomes are targeted into similar cytoplasmic inclusion bodies that contain the viral replication machinery [46]. In this case, however, it was suggested that the viral genomes reside in such inclusions whilst the virus dismantles the host innate immune response. This suggestion stems from the observation that the viral genomes are largely restricted to such inclusions following treatment of infected cells with interferon, linking an antiviral response to the presence of inclusions [46]. While we did not employ interferon treatment of cells in this study, we infected Vero cells (Figure 1E), which are known to have defects in interferon production. Consequently interferon production itself is not required for viral inclusion formation. We also tested a VSV mutant that is defective in host cell shut-off and is substantially less cytopathic. This virus contains a single mutation in the *M* gene that results in amino acid substitution M51R [47]. In cells infected with this rVSV-M51R virus, the viral replication machinery was also localized to inclusions (data not shown). Further experiments will therefore be required to examine the relationship between inclusion formation and the host response to infection.

Prior to the present study, experiments with two members of the *Rhabdoviridae*, rabies and VSV had reached different conclusions regarding the intracellular site of RNA synthesis. For rabies virus, RNA synthesis occurs at intracytoplasmic inclusions [30], whereas with VSV the viral RNA was found throughout the cytoplasm [32]. Working with VSV, we now show that RNA synthesis occurs throughout the cytoplasm prior to protein synthesis. This is clearly visualized in cells infected with wild-type VSV in which the input RNPs are visualized by staining with an antibody against N protein and the newly synthesized mRNA using an antibody against BrUTP (Figure S2). In this case, 100s of input viral particles are detected along with the RNA. Once viral protein synthesis occurs, RNA synthesis is redirected to inclusions - even in cells infected at very high multiplicity of infection (data not shown). Our analysis also demonstrates that RNA, which is synthesized at inclusions, is subsequently transported along microtubules to become distributed throughout the cytoplasm. This likely explains the previous observation [32] that VSV RNA was present throughout the cytoplasm.

### Microtubule-dependent RNA transport

The mechanism by which mRNA moves away from the inclusions involves transport along microtubules. Three pieces of evidence support this conclusion. First, the viral mRNAs are restricted to the inclusions following chemical depolymerization of microtubules with nocodazole. Second, the restriction of viral RNA to the inclusions is quickly released upon wash-out of nocodazole; and third, the viral mRNA are visualized along microtubules. The finding that viral RNA undergoes such directed transport is perhaps unsurprising as cellular and other viral RNA is frequently transported within the cytoplasm [48], [49]. For HIV, which synthesizes mRNA in the nucleus, the viral mRNA is transported in a microtubule-dependent manner to cytoplasmic ribosomes for translation [50]. For VSV mRNA, we do not know whether the RNA directly engages microtubule motor proteins or whether additional proteins are required to form an RNP complex that is transported by such motor proteins. Neither do we know which motor proteins are required for this process of RNA transport. It seems likely, however, that VSV has coopted the conventional cellular RNA transport machinery for this purpose. Ongoing experiments are aimed at determining how the virus ensures that its mRNAs are transported from their site of synthesis. An intriguing possibility is that the viral N protein facilitates this process. This is suggested by the fact that N protein is colocalized with the RNA on the surface of inclusions, but not once the RNA is transported away from the surface (Figure 6A).

By metabolic incorporation of [^35^S]-met, we also show that the depolymerization of microtubules diminished the rate of viral protein synthesis by 40% (Figure 8). Since we measured rates of protein synthesis rather than steady state levels, this suggests that ongoing transport of mRNA from inclusions facilitates translation. The rapid kinetics with which rates of viral protein synthesis are recovered following wash-out of nocodazole provides further support for the importance of an intact MT network for efficient translation. The finding that an intact MT network is required for efficient translation is in agreement with earlier work that demonstrated that VSV mRNAs initially associate with the cytoskeletal framework which facilitates their translation [51]. Our work now extends on this by providing evidence that the mRNAs are loaded onto the cytoskeletal network at the inclusions and this facilitates their translation. This suggests that the virus has evolved to make use of cellular transport pathways to ensure that the mRNAs are exported from the inclusions for translation.

VSV mRNAs are indistinguishable from cellular mRNA but they are translated efficiently in the presence of a profound host cell shut-off. Attempts to define a specific feature of the viral mRNA that facilitates this efficient translation have revealed that this is not an inherent property of the RNA. Rather, it reflects the ongoing and abundant transcription of viral mRNA from the genome [52]. Our data are consistent with and provide further support for this notion, as depolymerization of MT diminishes the rate of viral protein synthesis, even at 5 hpi when the intracellular pool of viral mRNA is high. We cannot eliminate the alternate possibility, that the structural integrity of the MT network plays a direct role in the translation of the viral mRNA. Since we did not observe a similar reduction in cellular protein synthesis following treatment with nocodazole (Figure 8A and C), we find this alternate explanation unlikely. It is currently unclear why there is a need for ongoing mRNA synthesis and transport to facilitate efficient viral protein synthesis.

### Formation of viral inclusions

In cells infected with VSV, we do not observe inclusion formation until infection has been established (Figure 1E). Combined with the fact that we can observe primary mRNA synthesis throughout the cytoplasm we favor the idea that a host response to infection results in the formation of inclusions. This may reflect a response to the production of viral RNA, and/or the N, P and L proteins. Since our data indicate that the viral mRNA are transported away from the inclusions for efficient translation, the viral proteins must in turn be targeted back to inclusions. Experiments with rabies virus suggest that the inclusions share some properties with cellular aggresomes [53], [54]. Aggresomes are cellular structures that recruit misfolded proteins by a dynein-dependent retrograde transport on microtubules [53], [54]. Such a transport mechanism might ensure the recruitment of viral proteins to inclusions. For rabies virus it has been demonstrated that P interacts with the LC8 dynein light chain [55], [56], and this may in turn bring L and N to such sites. Ongoing experiments are defining the mechanism by which VSV proteins are transported to such sites, as well as understanding whether the inclusions share properties with known cellular structures.

Compartmentalization of the viral replication machinery is a common property of many RNA viruses. Whether such compartments serve to facilitate RNA synthesis, shield the products of RNA synthesis from detection by innate immune sensors or are a consequence of a host response to infection is uncertain. Our work shows that for VSV, initial RNA synthesis occurs throughout the cytoplasm and that only in the presence of protein synthesis are inclusions formed. Such analysis of early events in infection, combined with the cost to viral protein synthesis of restricting mRNA to the inclusions lends some support to the idea that inclusions may reflect a host response to infection. Further work with VSV will likely help resolve the functional significance of such compartments in NNS RNA virus infected cells.

## Supporting Information

Figure S1 Localization of inclusions in relation to cellular membranes and organelles. CV-1 cells were infected with rVSV-eGFP-P (pseudo-colored in red) at an MOI of 3, fixed at 6 hpi and markers of the endoplasmic reticulum (calnexin), Golgi (GM130) and early endosomes (early endosomal antigen 1, EEA1) detected by immune fluorescence microscopy (green). Lysosomes and mitochondria were detected by incorporation of fluorescent lysotracker and mitotracker (green) into cells for 30 minutes prior to fixation. Cell nuclei were counterstained with DAPI (blue). Size bars = 10 µm.
(2.39 MB TIF)

Figure S2 Visualization of primary mRNA transcripts and input RNPs. BSR-T7 cells were treated with puromycin 15 minutes before infection with rVSV at an MOI of 500. At 2 hpi, cells were depleted of intracellular UTP and exposed to ActD and nocodazole. After 1 hour, cells were transfected with BrUTP. Viral RNA (green) as well as N protein (red) were detected 1h later by immuno staining prior to imaging by fluorescence microscopy. Two representative cells are shown. Size bar = 5 µm.
(0.51 MB TIF)

Video S1 A Z-series showing RFP-P protein localization at viral inclusions surrounded by RNA. BSR-T7 cells were infected with rVSV-RFP-P at an MOI of 3, and exposed to nocodazole at 4 hpi. Following a 1h incubation, cells were transfected with BrUTP, fixed 40 minutes later and the viral RNA (green) and RFP-P (red) visualized by confocal microscopy as described in methods. The video of a single representative cell shows combined Z-stacks (0.26 µm thickness) of images taken through the cell shown in Figure 6A.
(0.05 MB MOV)

Video S2 A three dimensional projection of viral RNA surrounding RFP-P inclusions. A 3D view of the cell shown in Video S1. The combined Z-stacks of images taken through the cell are rotated around the Y-axis. Grid lines represent 5 µm^2^.
(0.26 MB MOV)

Video S3 A Z-series showing N protein localization at viral inclusions surrounded by RNA. BSR-T7 cells were infected with rVSV at an MOI of 3 and exposed to nocodazole at 4 hpi. Following a 1h incubation, cells were transfected with BrUTP, fixed 40 minutes later and the viral RNA (green) and N (red) visualized by confocal microscopy as described in methods. The video of the two adjacent representative cells depicted in Figure 6A shows combined Z-stacks (0.26 µm thickness) of images through the cells.
(0.12 MB MOV)

Video S4 A three dimensional projection of viral RNA surrounding inclusions containing N protein. A 3D view of the cells shown in Video S3. The combined Z-stacks of images taken through the cells are rotated around the Y-axis. Grid lines represent 5 µm^2^.
(0.35 MB MOV)

Video S5 A Z-series showing L protein localization at viral inclusions surrounded by RNA. BSR-T7 cells were infected with rVSV at an MOI of 3 and exposed to nocodazole at 4 hpi. Following a 1h incubation, cells were transfected with BrUTP, fixed 40 minutes later and the viral RNA (green) and L (blue) visualized by confocal microscopy as described in methods. The video of a single representative cell shows combined Z-stacks (0.26 µm thickness) of images taken through the cell shown in Figure 6A.
(0.35 MB MOV)

Video S6 A three dimensional projection of viral RNA surrounding inclusions containing L protein. A 3D view of the cell shown in Video S5. The combined Z-stacks of images taken through the cell are rotated around the Y-axis. Grid lines represent 5 µm^2^.
(0.35 MB MOV)
