# Supplementary figures and images for: Protein Expression Redirects Vesicular Stomatitis Virus RNA Synthesis to Cytoplasmic Inclusions
Source: PLoS Pathog. 2010 Jun 24;6(6):e1000958. doi: 10.1371/journal.ppat.1000958 (PMC2891829; doi:10.1371/journal.ppat.1000958)

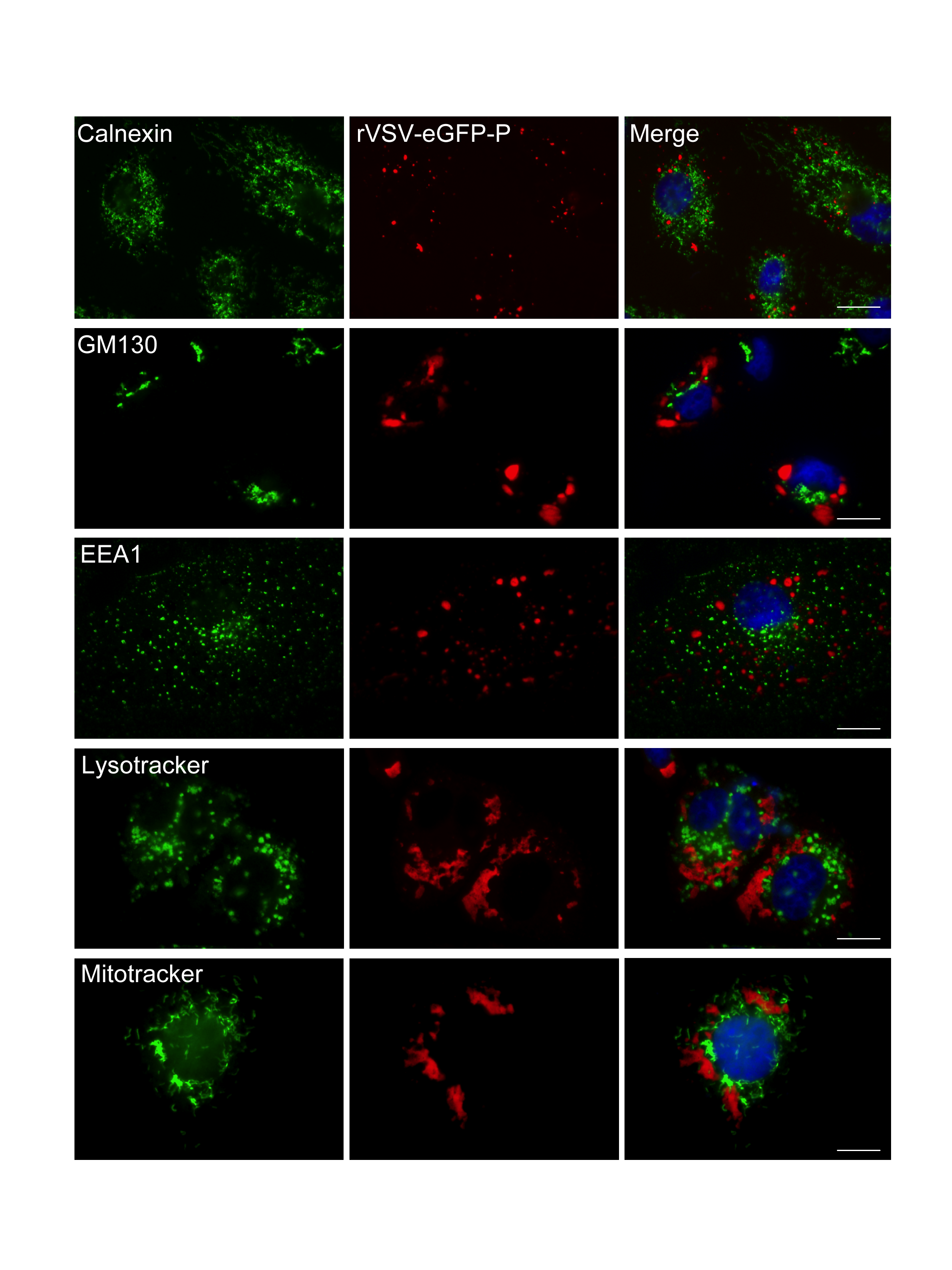

Supplement: Figure S1 — Localization of inclusions in relation to cellular membranes and organelles. CV-1 cells were infected with rVSV-eGFP-P (pseudo-colored in red) at an MOI of 3, fixed at 6 hpi and markers of the endoplasmic reticulum (calnexin), Golgi (GM130) and early endosomes (early endosomal antigen 1, EEA1) detected by immune fluorescence microscopy (green). Lysosomes and mitochondria were detected by incorporation of fluorescent lysotracker and mitotracker (green) into cells for 30 minutes prior to fixation. Cell nuclei were counterstained with DAPI (blue). Size bars = 10 µm. (2.39 MB TIF) [file ppat.1000958.s001.tif]

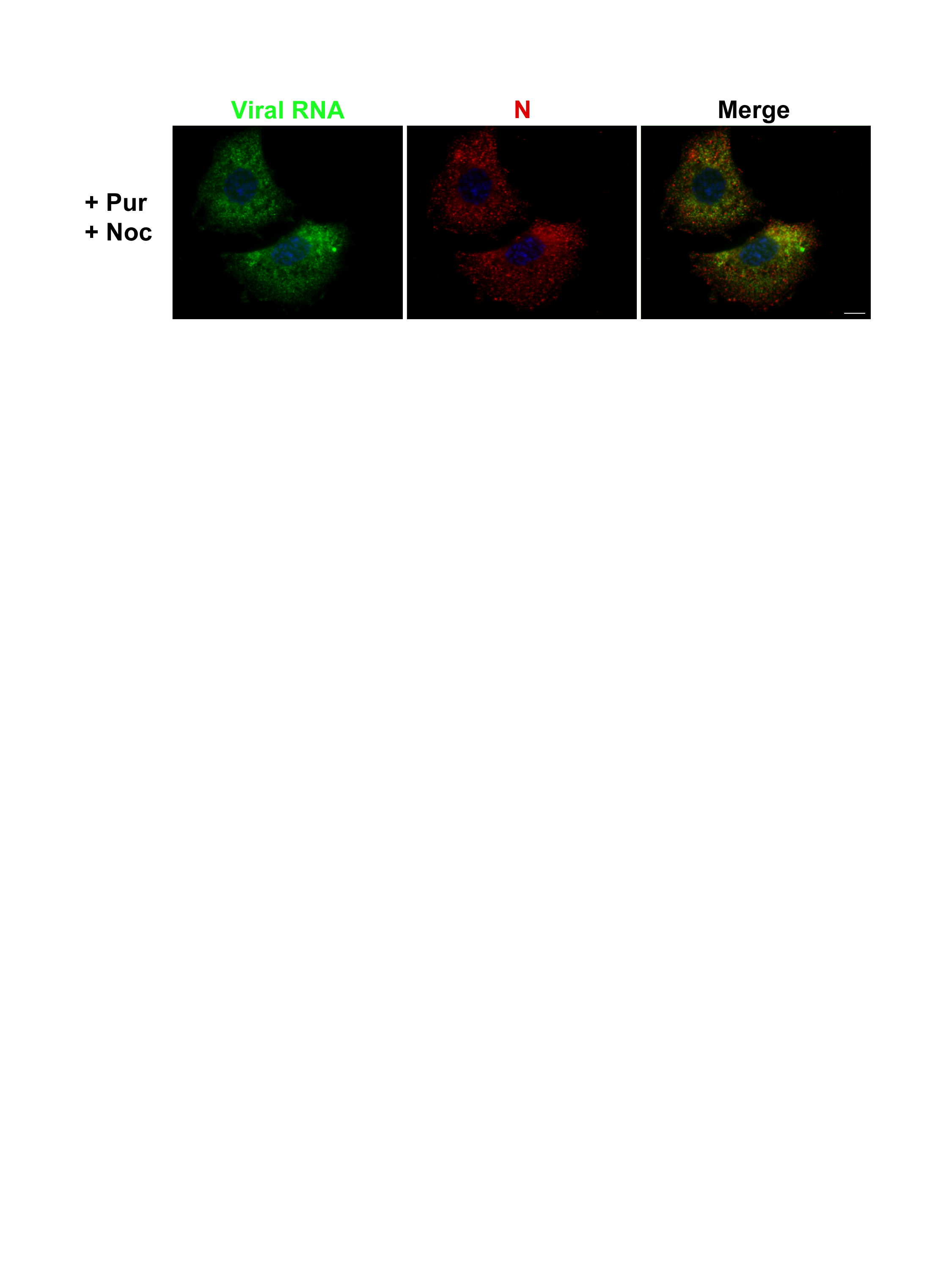

Supplement: Figure S2 — Visualization of primary mRNA transcripts and input RNPs. BSR-T7 cells were treated with puromycin 15 minutes before infection with rVSV at an MOI of 500. At 2 hpi, cells were depleted of intracellular UTP and exposed to ActD and nocodazole. After 1 hour, cells were transfected with BrUTP. Viral RNA (green) as well as N protein (red) were detected 1h later by immuno staining prior to imaging by fluorescence microscopy. Two representative cells are shown. Size bar = 5 µm. (0.51 MB TIF) [file ppat.1000958.s002.tif]
